# Study of seroprevalence of SARS‐CoV‐2 in Kazakhstan

**DOI:** 10.1017/S0950268823001085

**Published:** 2023-07-06

**Authors:** Mukhtar Kulimbet, Timur Saliev, Gulzhan Alimbekova, Dinara Ospanova, Kundyzay Tobzhanova, Dariga Tanabayeva, Baurzhan Zhussupov, Ildar Fakhradiyev

**Affiliations:** 1 B. Atchabarov Scientific-Research Institute of Fundamental and Applied Medicine, S.D. Asfendiyarov Kazakh National Medical University, Almaty, Republic of Kazakhstan; 2 Department of Public Health, Public Opinion Research Centre, Almaty, Republic of Kazakhstan; 3 Faculty of Medicine and Healthcare, Al-Farabi Kazakh National University, Almaty, Republic of Kazakhstan; 4 Department of Chemistry and Biology, Nazarbayev Intellectual School of Chemistry and Biology, Shymkent, Republic of Kazakhstan

**Keywords:** antibodies, asymptomatic, COVID‐19, SARS‐CoV‐2, seroprevalence

## Abstract

This study aimed to analyse the seroprevalence of SARS-CoV-2 in Kazakhstan. This is a cross-sectional study of adult population in Kazakhstan for the period from October 2021 to May 2022. For the study, 6 720 people aged 18 to 69 were recruited (from 17 regions). The demographic data were collected and analysed. Gender was evenly distributed (males 49.9%, females 50.1%). Women exhibited a higher seroprevalence than men (IgM 20.7% vs 17.9% and IgG 46.1% vs 41.5%). The highest prevalence of IgM was found in the age group of 30–39. However, the highest prevalence of IgG was detected in the age group of 60–69. The seroprevalence of IgG increased across all groups (from 39.7% in 18–29 age groups to 53.1% in 60–69 age groups). The odds for a positive test were significantly increased in older age groups 50–59 (*p* < 0.0001) and 60–69 (*p* < 0.0001). The odds of a positive test were 1.12 times higher in females compared to males (*p* = 0.0294). The odds for a positive test were significantly higher in eight regions (Astana, Akmola, Atyrau, Western Kazakhstan region, Kostanai, Turkestan, Eastern Kazakhstan region, and Shymkent) compared to Almaty city. The odds of a positive test were three times higher in Astana and the Western Kazakhstan region than in Almaty city. In urban areas, the odds of a positive test were 0.75 times lower than in rural areas (*p* < 0.0001). The study’s results showed an adequate level of seroprevalence (63%) that exceeds the essential minimum of herd immunity indicators in the country. There was significant geographic variability with a higher prevalence of IgG/IgM antibodies to SARS-CoV-2 in rural areas.

## Introduction

The rapid spread of the severe acute respiratory coronavirus 2 (SARS-CoV-2 / COVID-19) was declared a pandemic by the World Health Organization (WHO) in March 2020 [1]. In Kazakhstan, the first cases of coronavirus infection were recorded in mid-March 2020 [2]. A high incidence and mortality rate from COVID-19-like pneumonia was detected [3]. According to WHO statistics (early 2023), there were 1 496 390 confirmed cases of COVID-19 with 19 065 deaths [4]. Apart from the adult population [2], cases of COVID-19 infection were recorded in children too [5]. In addition, cases of outbreaks were also observed in the country’s different regions as well as in healthcare organisations [6, 7].

The actual statistics on confirmed cases and deaths could help monitor and analyse the evolution of the disease. However, such information is not ideal to estimate the proportion of the population infected. It is an important indicator for the analysis of the COVID-19 epidemiological situation and the development of policy strategies [8].

Seroprevalence studies are of paramount importance for estimating the proportion of the population that has already developed antibodies to the virus and could potentially be protected from subsequent infection [9]. The antibodies are a marker of complete or partial immunity. The information about antibodies could help to estimate the proportion of the population that remains susceptible to the virus [10]. A study was previously conducted to assess the seroprevalence of SARS-CoV-2 among the population of Kazakhstan in the period from 2020 to 2021 [11]. Nonetheless, this study was based on the analysis of antibodies to the SARS-CoV-2 virus in self-reported individuals, which cannot truly represent the level of seroprevalence of the entire population as a whole. Other studies conducted on the territory of Kazakhstan on the prevalence of antibodies to COVID-19 infection were limited to the local geographical location [12], and were based on a rather small sample size [13]. For example, one retrospective cohort study examined data from patients with laboratory-confirmed cases of COVID-19 [14]. However, the study period was short, which included only one-third of all laboratory-confirmed cases of COVID-19 in March–April 2020 (in Kazakhstan). There was the heterogeneous quality of medical record data, and the limited availability and lack of data for some variables. Moreover, there were other factors and weak sides, including the deficiency of information on performance characteristics of COVID-19 diagnostic tests across the country, small sample size, and insufficient capacity to assess associations of viral diversity with clinical outcomes [14].

One of the main means of combating a pandemic is the availability of an effective vaccine to control the spread of a deadly virus. The level of vaccination affects the establishment of an immune response as a preventive measure for reinfection [15]. An earlier study focused on the analysis of a vaccination survey in Kazakhstan [16]. However, this study had limitations due to a small sample size and some errors in the study’s sampling method. As of 31 December 2022, a total of 33 496 177 vaccine doses were administered in Kazakhstan according to WHO [4]. Nevertheless, there are no data on SARS-CoV-2 seroprevalence based on vaccination status.

Serologic testing is the best tool to determine the spread of infectious disease, especially when there are asymptomatic cases or incomplete identification of symptomatic individuals [17]. In this regard, given the lack of comprehensive epidemiological data at the national level, all of the above-mentioned facts indicate the need for a large-scale study of seroprevalence for the SARS-CoV-2 virus. Thus, the purpose of this study was to determine the seroprevalence of SARS-CoV-2 in Kazakhstan in the period from October 2021 to May 2022.

## Methods

### Ethical issues

The study was approved by the Local Ethics Committee of the S.D. Asfendiyarov Kazakh National Medical University, Almaty, Republic of Kazakhstan (protocol of the Local Ethics Commission No. 12 (118) of 28.09.2021). Moreover, the study was registered with ClinicalTrials.gov (NCT05122832).

### Study design and population

This is a cross-sectional study of the adult population in Kazakhstan for the period from October 2021 to May 2022. We recruited 6 720 people aged 18 to 69 throughout Kazakhstan (17 regions including the cities of Almaty, Astana, and Shymkent). Participation in the study was completely voluntary.

### Study setting

Kazakhstan is located in Central Asia. It is administratively divided into 14 regions with 177 districts and cities. In addition, Kazakhstan has three cities of ‘republican significance’: Astana, (the capital city, previously known as ‘Nur-Sultan’), Almaty, the former capital city, and Shymkent, the third largest city in KZ. In general, urban areas are considered as the town or city, and rural areas as the district. The country’s population is around 20 million people, and the population density is 6 people per square kilometre. The majority of inhabitants reside in urban areas [18].

### Sampling

We used a weighted, multistage, cluster sampling method and included eight groups with a division into four age groups – 18-29 years, 30–44 years, 45–59 years, 60–69 years – as well as with stratification by sex (men and women) in each age group. The study sample size was determined using the WHO’s special STEPS tool (sample_size_calculator Excel format) via the following methodology:

Probability value for 95% confidence interval - 1.96;

Estimated prevalence of the risk factors - 0.5;

Margin of error - 0.05;

Design effect −1.5;

Anticipated response rate - 70%.

The preliminary calculation resulted in the sample size of n = 6585.

In the first stage, we selected the primary sampling units: districts and cities. The primary sampling units (clusters) were proportionally selected among all economic regions. Information about districts and cities (Almaty, Astana, and Shymkent cities) and all 14 regions was received from the Bureau of National Statistics, Agency for Strategic Planning and the Republic of Kazakhstan [18].

In the second stage, we selected the secondary sampling units (SSU) of Primary Health Care facilities (PHC), which provide medical care for the local population. For a selection of SSUs, we used data from the Republican state enterprise on the right of economic management, the ‘Republican Centre for Healthcare Development’ of the Ministry of Health of the Republic of Kazakhstan [18]. A register of PHC facilities was obtained with an indication of the number of people served. SSUs were selected by random sampling method and with a probability proportional to the number of populations served in each PHC facility.

In the third stage, we selected the tertiary sampling units: households and respondents. The size of households per PHC facility was calculated using the following formula:

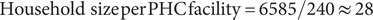



Then we calculated the final total sample size: Final Total Sample Size = 240 × 28 = 6720.

For the selection of households, a list of households served by chosen PHC facilities was obtained. Households were randomly selected from each facility using the Randhold.xls tool to participate in the study. The final selection of respondents aged 18–69 from each selected household was carried out using the Kish method. This was done using a special methodology, including a random selection of the respondent depending on the sex and age of all residents of the household that meet the criteria for inclusion in this study.

### Covariates

The zero-positivity rates were evaluated as ≥1.4 for IgG and ≥ 1 for IgM. Age groups were categorised as follows: 18–29, 30–39, 40–49, 50–59, 60–69. Gender was categorised as male and female. Data on place of residence was categorised as urban and rural. The regional data was represented as all 14 regions and 3 cities. Ethnicity was categorised as Kazakh, Russian, Uzbek, Ukraine, Uighur, Tatar, and others.

According to the questionnaire used, the study participants were asked questions with multiple choice answers: Have you ever had COVID-19? (answers: yes, no); How were you diagnosed with COVID-19 infection? (answers: PCR method, rapid test, IgG test, IgM test, X-ray / computed tomography, clinical methods, and other methods); Have you been vaccinated against COVID-19? (answers: yes, no, I don’t know); What type of vaccine did you receive? (answers: Sputnik V, QazVac, Hayat-Vax, Corona Vac, Pfizer-Biontech, AstraZeneca, Moderna, other); How many doses of vaccine did you receive? (one, two, I don’t know).

### Laboratory methods

Seroprevalence was determined using a commercially available Abbott Alinity™ automated immunoassay analyser SARS-Test for IgM and IgM antibodies to SARS-CoV-2 [19].

The delivery of laboratory tests for antibodies to the SARS-CoV-2 virus was carried out in the branches of the private largest medical laboratories located in all regions of Kazakhstan. The interpretation of the test for the qualitative determination of IgM to the SARS Cov-2 virus was done as follows: <1.0 s/c -negative; >1.0 s/s is positive. Analytical reproducibility-2.9%. Positive consistency: 8–14 days after the onset of symptoms 79.56%, 15–30 days 91.26%, and more than 31 days 94.74%. The predictive value of a positive result is 92.07%. The predictive value of a negative result is 99.82%.

The interpretation of the test for the qualitative determination of IgG in the SARS-Cov-2 virus was done as follows: <1.4 s/c -negative; >1.4 s/c is positive. Analytical reproducibility-5.9%. Positive agreement: 8–14 days after the onset of symptoms 86.36%, 15–30 days 100%. Negative consistency: 99.63%.

Our survey protocol was based on the WHO protocol for testing for antibodies to COVID-19 at the population level [20, 21].

### Statistical analysis

The statistical analyses were processed via SAS OnDemand for Academics (release 3.81, Carry, NC, USA). Categorical variables were expressed as frequency and percent. We used the Chi-square test and Fisher’s exact test to assess differences and associations between categorical variables. Ninety‐five percent confidence intervals (CIs) were calculated. The data were weighted taking into account the sex and age distribution of the population of Kazakhstan. Continuous variables were expressed as mean and standard deviation (SD) and were analysed using a one-way analysis of variance. The Kruskal–Wallis test was used for comparison of non-normally distributed continuous variables and presented as median (interquartile range). We used logistic regression to estimate odds ratios (ORs) and 95% confidence intervals (CIs). The alpha level was set up at 0.05, considering it statistically significant.

## Results

We analysed IgG and IgM antibody levels to SARS-CoV-2 in 6720 healthy persons from 14 regions and 3 major cities of Kazakhstan. The overall median age of participants was 39 years. Table 1 shows the demographic characteristics of the participants.

**Table 1.** Demographic characteristics of the participants

Characteristics	Count	Percent
Age in years, median	6720	39.00
Age		
18–29	1728	25.71
30–39	1688	25.12
40–49	1310	19.49
50–59	1159	17.25
60–69	835	12.43
Gender		
Male	3365	50.07
Female	3355	49.93
Education in years, median	6720	14.00
Place of residence		
Urban	4401	65.49
Rural	2319	34.51
Occupation		
State employee	853	12.69
Private sector worker	2584	38.45
budget employee	820	12.2
Entrepreneur	552	8.21
Agricultural worker	50	0.74
Student	291	4.33
Housewife	501	7.46
Retiree	648	9.64
unemployed (able to work)	334	4.97
unemployed (unable to work)	58	0.86
Refused to answer	29	0.43
Region		
Nur-Sultan c.	448	6.67
Almaty c.	560	8.33
Akmola r.	336	5.00
Aktobe r.	336	5.00
Almaty r.	560	8.33
Atyrau r.	336	5.00
Western Kazakhstan r.	224	3.33
Zhambyl r.	448	6.67
Karaganda r.	448	6.67
Kostanai r.	336	5.00
Kyzylorda r.	336	5.00
Mangistau r.	336	5.00
Turkestan r.	560	8.33
Pavlodar r.	336	5.00
North Kazakhstan r.	224	3.33
Eastern Kazakhstan r.	448	6.67
Shymkent c.	448	6.67
Ethnicity		
Kazakh	4374	65.09
Russian	1550	23.07
Uzbek	202	3.01
Ukraine	110	1.64
Uighur	37	0.55
Tatar	117	1.74
Others	323	4.81
Refused to answer	7	0.1

More than half of the participants were in the 18–29 (25.7%) and 30–39 (25.1%) age groups. Gender was almost evenly distributed (males 49.9%, females 50.1%). Participants who live in urban areas accounted for 65.5% (n = 4401). Almaty city (8.3%), Almaty region (8.3%), and Turkestan region (8.3%) had more participants (560 each) than other regions. The majority of the participants were Kazakhs (65.1%) and Russians (23.1%). The median education was 14 years. Most of the participants were private sector workers (38.45%), state employees (12.69%), and budget employees (12.2%) (Table 1).

**Figure 1.**
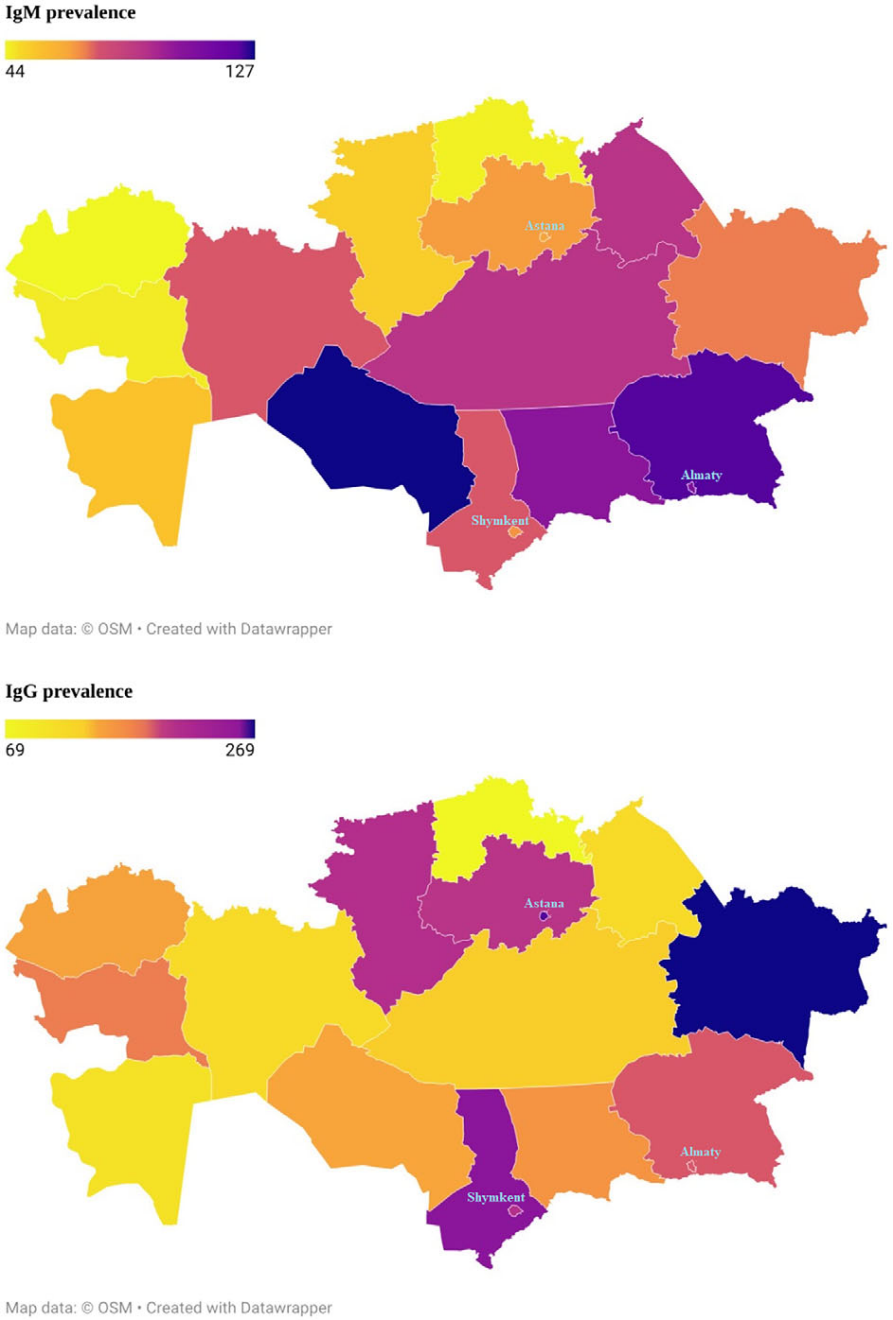
The prevalence of IgM and IgG antibodies to the SARS Cov-2 virus in the Republic of Kazakhstan for the period October 2021 to May 2022.

The results showed that the women had a higher seroprevalence than men (IgM 20.7% vs 17.9% and IgG 46.1% vs 41.5%). The highest prevalence of IgM was found in the age group of 30–39. However, the highest prevalence of IgG was found in the age group of 60–69. The seroprevalence of IgG was found to increase with age (from 39.7% in the 18–29 age group to 53.1% in the 60–69 age group). Rural areas had more seroprevalence of IgG than urban areas. The higher seroprevalence of IgM was found in the Kyzylorda region (37.8%); in contrast, the lowest seroprevalence was found in the Turkestan region (12.9%). The seroprevalence of IgM in the majority of regions ranged between 15% and 22%. The higher seroprevalence of IgG was found in the Western Kazakhstan region (64.7%); in contrast, the lowest seroprevalence was found in three regions: Karaganda (30.6%), North Kazakhstan region (30.8%), and Mangistau (30.9%) (Figure 1). In terms of occupation, the prevalence of IgM was higher among agriculture workers (28%) and those who refused to answer this question (27.59%). A higher prevalence of IgG was observed among retired participants (52.47%) and who refused to answer this question (48.28%).

The seroprevalence of IgG in the majority of regions ranged between 30% and 47%. The range of seroprevalence in IgM by main ethnic groups was from 14.4% to 21.8%. The seroprevalence in IgM in the dominant ethnic groups such as Kazakhs and Russians were 19.2% and 20.2%, respectively. The range of seroprevalence in IgG by ethnic groups was from 36.8% to 49.1%. The seroprevalence in IgG in the major ethnic groups such as Kazakhs and Russians were 44.1% and 44.2%, respectively (Table 2).

**Table 2.** Prevalence of antibodies to SARS‐CoV‐2

Characteristics	Total N (%)	IgM Positive % (n)	IgG Positive % (n)
Age			
18–29	1728 (25.71)	16.03 (277)	39.70 (686)
30–39	1688 (25.12)	21.21 (358)	41.88 (707)
40–49	1310 (19.49)	19.92 (261)	42.29(554)
50–59	1159 (17.25)	20.19 (234)	47.63 (552)
60–69	835 (12.43)	20.00 (167)	53.05 (443)
Gender			
Male	3365 (50.07)	17.92 (603)	41.52 (1397)
Female	3355 (49.93)	20.69 (694)	46.05 (1545)
Occupation			
State employee	853 (12.69)	20.05 (171)	40.68 (347)
Private sector worker	2584 (38–45)	18.07 (467)	41.91 (1083)
budget employee	820 (12.2)	19.15 (157)	46.46 (381)
Entrepreneur	552 (8.21)	20.47 (113)	43.30 (239)
Agricultural worker	50 (0.74)	28.00 (14)	42.00 (21)
Student	291 (4.33)	15.12 (44)	37.80 (110)
Housewife	501 (7.46)	21.76 (109)	45.51 (228)
Retiree	648 (9.64)	19.91 (129)	52.47 (340)
unemployed (able to work)	334 (4.97)	22.46 (75)	45.51 (152)
unemployed (unable to work)	58 (0.86)	17.24 (10)	46.55 (27)
Refused to answer	29 (0.43)	27.59 (8)	48.28 (14)
Place of residence			
Urban	4401 (65.49)	19.02 (837)	41.58 (1830)
Rural	2319 (3451)	19.84 (460)	47.95 (1112)
Region			
Nur-Sultan c.	448 (6.67)	14.73 (66)	59.82 (268)
Almaty c.	560 (8.33)	18.93 (106)	33.57 (188)
Akmola r.	336 (5.00)	19.94 (67)	58.33 (196)
Aktobe r.	336 (5.00)	21.43 (72)	34.23 (115)
Almaty r.	560 (8.33)	22.50 (126)	33.57 (188)
Atyrau r.	336 (5.00)	13.69 (46)	52.98 (178)
Western Kazakhstan r.	224 (3.33)	19.64 (44)	64.73 (145)
Zhambyl r.	448 (6.67)	22.54 (101)	35.04 (157)
Karaganda r.	448 (6.67)	20.09 (90)	30.58 (137)
Kostanai r.	336 (5.00)	15.18 (51)	61.01 (205)
Kyzylorda r.	336 (5.00)	37.80 (127)	42.56 (143)
Mangistau r.	336 (5.00)	16.37 (55)	30.95 (104)
Turkestan r.	560 (8.33)	12.86 (72)	46.79 (262)
Pavlodar r.	336 (5.00)	26.79 (90)	34.23 (115)
North Kazakhstan r.	224 (3.33)	20.09 (45)	30.80 (69)
Eastern Kazakhstan r.	448 (6.67)	15.85 (71)	60.04 (269)
Shymkent c.	448 (6.67)	15.18 (68)	45.31(203)
Ethnicity			
Kazakh	4374 (65.09)	19.16 (838)	44.06 (1927)
Russian	1550 (23.07)	20.19 (313)	44.19 (685)
Uzbek	202 (3.01)	14.36 (29)	39.60 (80)
Ukraine	110 (1.64)	21.82 (24)	49.09 (54)
Uighur	37 (0.55)	21.62 (8)	43.24 (16)
Tatar	117 (1.74)	18.80 (22)	36.75 (43)
Others	323 (4.81)	18.89 (61)	40.56 (131)
Refused to answer	7 (0.10)	28.57 (2)	85.71 (6)

Table 3 includes the questions related to the diagnosis and vaccination of participants by IgM antibody to SARS-CoV-2. Positive cases were more likely to have had SARS-CoV-2 (30.9%, *p* < 0.0001). Participants with a positive IgM antibody test were more likely to have been diagnosed with a PCR test than participants with a negative IgM antibody test (60.2% vs 55.7%, *p* = 0.0384). Those with negative IgM antibody tests were more likely to have received a vaccine for coronavirus than those with positive IgM antibody tests. However, it was not statistically significant (*p* = 0.2107). The patients with negative cases were more likely to have taken the Sputnik V vaccine (compared to positive cases) (77.1% vs 74.9%, *p* < 0.0001).

**Table 3.** Characteristics of IgM

	Total	Positive	Negative	*p*-value
SARS Cov-2				<0.0001
Yes	1680 (25.00)	402 (30.99)	1278 (23.57)	
No	4751 (70.70)	848 (65.38)	3903 (71.97)	
Do not know	289 (4.30)	47 (3.62)	242 (4.46)	
Diagnosis confirmed				0.0384
PCR	954 (56.79)	242 (60.20)	712 (55.71)	
Express test	24 (1.43)	5 (1.24)	19 (1.49)	
IgG test	40 (2.38)	12 (2.99)	28 (2.19)	
IgM test	15 (0.89)	1 (0.25)	14 (1.10)	
X-ray/CT	181 (10.77)	53 (13.18)	128 (10.02)	
Clinical	207 (12.32)	40 (9.95)	167 (13.07)	
Other	259 (15.42)	49 (12.19)	210 (16.43)	
Vaccine				0.2107
Yes	4758 (70.80)	893 (68.85)	3865 (71.27)	
No	1950 (29.02)	401 (30.92)	1549 (28.56)	
Do not know	12 (0.18)	3 (0.23)	9 (0.17)	
Type of Vaccine				<0.0001^a^
Sputnik V	3648 (76.67)	669 (74.92)	2979 (77.08)	
QazVac	328 (6.89)	55 (6.16)	273 (7.06)	
Hayat-Vax	359 (7.55)	72 (8.06)	287 (7.43)	
Corona Vac	153 (3.22)	34 (3.81)	119 (3.08)	
Pfizer-Biontech	96 (2.02)	17 (1.90)	79 (2.04)	
AstraZeneca	2 (0.04)	0	2 (0.05)	
Moderna	1 (0.02)	0	1 (0.03)	
Other	171 (3.59)	46 (5.15)	125 (3.23)	
Number of vaccine doses				0.5013
One	264 (5.55)	53 (5.94)	211 (5.46)	
Two	4472 (93.99)	834 (93.39)	3638 (94.13)	
Do not know	22 (0.46)	6 (0.67)	16 (0.41)	

a Fisher’s exact test.

Table 4 includes the questions related to the diagnosis and vaccination of participants by IgG antibody to SARS-CoV-2. Positive cases were more likely to have had SARS-CoV-2 (31.8%, *p* < 0.0001). Participants with a positive IgG antibody test were more likely to have been diagnosed with a PCR test than participants with a negative IgG antibody test (62.4% vs 49.7%, *p* < 0.0001). Those with negative IgG antibody tests were more likely to have received a vaccine for coronavirus than those with positive IgG antibody tests (72.1% vs 69.1%, *p* = 0.0287). The patients with negative cases were more likely to have taken the Sputnik V vaccine (compared to positive cases) (81.8% vs 69.8%, *p* < 0.0001).

**Table 4.** Characteristics of IgG

	Total	Positive	Negative	*p*-value
SARS Cov-2				<0.0001
Yes	1680 (25.00)	936 (31.82)	744 (19.69)	
No	4751 (70.70)	1878 (63.83)	2873 (76.05)	
Do not know	289 (4.30)	128 (4.35)	161 (4.26)	
Diagnosis confirmed				<0.0001
PCR	954 (56.79)	584 (62.39)	370 (49.73)	
Express test	24 (1.43)	12 (1.28)	12 (1.61)	
IgG test	40 (2.38)	22 (2.35)	18 (2.42)	
IgM test	15 (0.89)	7 (0.75)	8 (1.08)	
X-ray/CT	181 (10.77)	109 (11.65)	72 (9.68)	
Clinical	207 (12.32)	76 (8.12)	131 (17.61)	
Other	259 (15.42)	126 (13.46)	133 (17.88)	
Vaccine				0.0287
Yes	4758 (70.80)	2034 (69.14)	2724 (72.10)	
No	1950 (29.02)	902 (30.66)	1048 (27.74)	
Do not know	12 (0.18)	6 (0.20)	6 (0.16)	
Type of Vaccine				<0.0001^a^
Sputnik V	3648 (76.67)	1420 (69.81)	2228 (81.79)	
QazVac	328 (6.89)	193 (9.49)	135 (4.96)	
Hayat-Vax	359 (7.55)	194 (9.54)	165 (6.06)	
Corona Vac	153 (3.22)	93 (4.57)	60 (2.20)	
Pfizer-Biontech	96 (2.02)	44 (2.16)	52 (1.91)	
AstraZeneca	2 (0.04)	0	2 (0.07)	
Moderna	1 (0.02)	1 (0.05)	1 (0.05)	
Other	171 (3.59)	89 (4.38)	82 (3.01)	
Number of vaccine doses				
One	264 (5.55)	134 (6.59)	130 (4.77)	0.0157
Two	4472 (93.99)	1893 (93.07)	2579 (94.68)	
Do not know	22 (0.46)	7 (0.34)	15 (0.55)	

a Fisher’s exact test.

Table 5 illustrates the logistic regression of the influencing factors for the IgG antibody positive rate. By increasing age, the IgG antibody positive rate increases. The odds for a positive test were significantly increased in older age groups – 50–59 (*p* < 0.0001) and 60–69 (*p* < 0.0001). The odds of a positive test were 1.12 times higher in females compared to males (*p* = 0.0294). The odds for a positive test were significantly higher in eight regions (Astana, Akmola, Atyrau, Western Kazakhstan region, Kostanai, Turkestan, Eastern Kazakhstan region, and Shymkent) compared to Almaty city. The odds of a positive test were three times higher in Astana and Eastern Kazakhstan region than in Almaty city. In urban areas the odds of a positive test were 0.75 times lower than in rural areas (*p* < 0.0001). The odds of a positive test were significantly higher among private sector workers (*p* = 0.0448), housewives (*p* = 0.0336), and the unemployed (able to work) (*p* = 0.0274) compared to state employees.

**Table 5.** Binary logistic regression of the influencing factors for IgG antibody positive rate

Variables	IgG antibody positive rate (%)	P-value	OR	95% CI
Age				
18–29^a^	39.70	–	–	–
30–39	41.88	0.2186	1.09	0.95–1.26
40–49	42.29	0.2525	1.09	0.94–1.27
50–59	47.63	<.0001	1.37	1.16–1.59
60–69	53.05	<.0001	1.70	1.42–2.02
Education		0.4484	0.99	0.97–1.01
Gender				
Male^a^	41.52	–	–	–
Female	46.05	0.0294	1.12	1.01–1.24
Occupation				
State employee^a^	40.68	–	–	–
Private sector worker	41.91	0.0448	1.185	1.004–1.39
Budget employee	46.46	0.0752	1.202	0.98–1.47
Entrepreneur	43.30	0.0687	1.234	0.98–1.55
Agricultural worker	42.00	0.9702	0.989	0.54–1.79
Student	37.80	0.5156	1.106	0.82–1.49
Housewife	45.51	0.0336	1.293	1.02–1.64
Retiree	52.47	0.2347	1.198	0.89–1.61
Unemployed (able to work)	45.51	0.0274	1.351	1.03–1.76
Unemployed (unable to work)	46.55	0.2501	1.382	0.79–2.39
Refused to answer	48.28	0.1462	1.762	0.82–3.78
Region				
Almaty c.^a^	33.57	–	–	–
Nur-Sultan c.	59.82	<.0001	3.09	2.38–4.00
Akmola r.	58.33	<.0001	2.38	1.78–3.17
Aktobe r.	34.23	0.7770	0.96	0.72–1.28
Almaty r.	33.57	0.1182	0.81	0.62–1.06
Atyrau r.	52.98	<.0001	2.04	1.53–2.70
Western Kazakhstan r.	64.73	<.0001	3.23	2.32–4.50
Zhambyl r.	35.04	0.4938	0.91	0.69–1.20
Karaganda r.	30.58	0.1469	0.82	0.63–1.07
Kostanai r.	61.01	<.0001	2.80	2.11–3.73
Kyzylorda r.	42.56	0.1207	1.56	0.94–1.68
Mangistau r.	30.95	0.2588	0.84	0.63–1.13
Turkestan r.	46.79	0.0032	1.47	1.14–1.91
Pavlodar r.	34.23	0.8390	0.97	0.73–1.30
North Kazakhstan r.	30.80	0.0971	0.75	0.53–1.05
Eastern Kazakhstan r.	60.04	<.0001	2.68	2.06–3.49
Shymkent c.	45.31	<.0001	1.71	1.32–2.21
Place of residence				
Rural^a^	41.58	–	–	–
Urban	47.95	<.0001	0.75	0.66–0.85

a Reference.

## Discussion

This cross-sectional study is the first large-scale research on COVID-19 seroprevalence among the entire adult population of 17 regions of Kazakhstan. The selection of study participants was carried out according to a special methodology, including random selection of the respondent depending on the sex and age of all household residents who meet the inclusion criteria in this study. The study period covered October 2021–May 2022.

According to the WHO recommendations, monitoring changes in seroprevalence over time is critical at the start of an epidemic to predict its dynamics and plan an adequate public health response [20].

In fact, antibody titters may decrease over time [9]. Currently, the persistence of antibodies post COVID-19 infection is not precisely known. Therefore, the period of infection in participants who tested positive for COVID-19 antibodies is not possible to indicate in this study. In the period July–August 2021I, the Delta strain of coronavirus infection was spreading in Kazakhstan [22]. The study results indicate that the prevalence of IgM was relatively high in participants aged 30–39 years, while IgG prevailed in participants aged 60–69 years.

In our study, in terms of IgM and IgG, women had higher seroprevalence rates compared with men. Moreover, according to the results of the logistic regression analyses, the probability of having IgG to the SARS-CoV-2 virus is 1.12 times higher in women compared to men (*p* = 0.0294). This finding is consistent with the results of other studies [23]. However, many studies do not confirm the existence of a relationship between the prevalence of antibodies to COVID-19 and gender [9, 24].

The analysis revealed that SARS-CoV-2 seroprevalence varied across geographic regions, for instance, between urban and rural areas. In fact, urban areas are denser populated than rural areas, which may affect the faster spread of the virus [25]. We observed that IgG seroprevalence was higher in rural areas than in urban areas. Nevertheless, earlier studies of COVID-19 cases in 2020 showed a prevalence of infection among urban residents [2]. It may have been due to lower detection rates and a shortage of diagnostic tests at the beginning of the COVID-19 pandemic in rural areas. This finding has been also supported by the results of other studies, indicating that a high population density (in large urban areas) forced the use of strict non-pharmacological measures, such as quarantine and social distancing practices. It led to the reduction of the spread of infection among the urban population [26]. It encompasses wearing masks as an effective and preventive public health tool against the spread of the virus. In turn, some studies have found a lower frequency of mask use in public places in rural areas compared to urban ones [27]. Moreover, in most rural areas, there is no public effective health infrastructure, which is often under-resourced, including diagnostic service [28]. This fact can also impact the understanding of the true prevalence of antibodies to the SARS-CoV-2 virus.

A higher IgM seroprevalence was detected in the Kyzylorda region (37.8%), whilst the lowest seroprevalence was found in the Turkestan region (12.9%). A higher IgG seroprevalence was detected in the Western region (64.7%). At the same time, the lowest seroprevalence was found in three regions: Karaganda (30.6%), Northern Kazakhstan (30.8%), and Mangistau (30.9%).

According to the answers of the respondents, the presence of IgM and IgG antibodies in the blood serum confirmed the incidence of COVID-19 (according to the anamnesis) (*p* < 0.0001).

Our findings indicate that the level of positive IgG antibodies increased over time. A statistically significant relationship was determined with a positive test result for the presence of IgG to the SARS-CoV-2 virus and ages 50–59 years and 60–69 years (*p* < 0.0001). The chances of a positive test result were significantly higher in eight regions (Astana, Akmola region, Atyrau, West Kazakhstan region, Kostanay, Turkestan, East Kazakhstan region, Shymkent) compared to Almaty city. The number of positive tests was three times higher in the city of Astana and the Western Kazakhstan region than in the city of Almaty.

Herd immunity occurs when a certain subset of a population is immune to a given infectious disease, reducing the likelihood that the disease will be transmitted from one individual to another, thereby helping to protect the entire population from that disease [29]. On 1 February 2021, vaccination against COVID-19 began in Kazakhstan using a vaccine against the Gam-COVID-Vac viral vector (‘Sputnik V’) produced in Russia [30]. In our study, participants who tested negative for IgG antibodies were more likely to receive the coronavirus vaccine than those who tested positive for IgG antibodies (*p* = 0.0287). Moreover, respondents with negative IgM and IgG results for the SARS-CoV-2 virus were relatively more likely to confirm vaccination with the Sputnik V vaccine than positive cases (77.1% versus 74.9%, *p* < 0.0001).

The use of serology as a tool to fight a pandemic could move from a diagnostic tool to a tool for prioritising vaccination. For example, seronegative individuals may be prioritised over seropositive individuals in order to achieve better uptake of vaccinations and obtain ‘herd immunity’ earlier [31].

Our study highlights the importance of repeat sero-surveillance as a strategy to monitor and study how infection patterns evolve over time. The results of this study can subsequently be used as data for comparison with seroprevalence studies in subsequent periods, which will help evaluate the effectiveness of preventive measures to maintain a stable epidemiological situation.

## Conclusions

The study’s findings showed that seroprevalence levels at 63%, which are over the required minimum for nation herd immunity, are acceptable. There was a substantial geographic difference, with rural areas having a greater incidence of IgG/IgM antibodies to SARS-CoV-2. The results indicated how critical natural population immunity is for the development of a SARS-CoV-2 policy.

## Study limitations

Study samples were collected in proportion to the distribution of participants by region and age. However, data such as clinical symptoms and confirmation of SARS-CoV-2 infection were not analysed in this study. In addition, the study was conducted over a certain period of time, thus the results can only reflect the situation at that period. Furthermore, since the nature of this study was cross-sectional, we will not be able to see the dynamics of seroprevalence over time. A longitudinal study could provide more information on how seroprevalence changes over time and how this is related to the level of population immunity to the SARS- CoV-2.

## Data Availability

All available data were indicated within manuscript text.
